# Reproductive outcomes of patients with T-Shaped and septate uterus following metroplasty: an observational study

**Published:** 2020-03-27

**Authors:** R Pabuccu, EG Pabuccu, V Gomel

**Affiliations:** Ufuk University School of Medicine, Department of Obstetrics and Gynecology, Ankara, Turkey;; Centrum Clinic IVF Center, Ankara, Turkey;; University of British Columbia, Department of Obstetrics and Gynecology, Vancouver, Canada.

**Keywords:** Hysteroscopy, infertility, metroplasty, uterine septum, T-shaped uterus

## Abstract

**Introduction:**

Uterine malformations are common and may contribute to infertility and adverse pregnancy outcomes. After an accurate diagnosis, correcting the abnormal uterine morphology is the main goal to optimize reproductive outcomes. The principal objective of this study was to assess the impact of metroplasty for T-shaped (U1a) and septate uteri (U2) on live birth rates in infertile patients.

**Methods:**

This was a prospective observational study of infertile women with either U1a or U2 uterine anomaly. Patients with unexplained infertility and repeated (IVF/ICSI) failure were included. Hysteroscopic metroplasty was performed by a single experienced surgeon. Fertility outcomes of all cases were evaluated prospectively evaluated. The main outcome parameter was a live birth rate either achieved spontaneously or with assisted conception.

**Results:**

A total of 48 patients were included in U1a group and bilateral longitudinal uterine-lateral wall incision was carried out. A total of 63 patients were included in the U2 group and septum incision was carried out, 60 out of these 63 patients with U2 uterine anomaly required further lateral wall incision during septoplasty. During the first 12 months following surgery, nearly half of the patients in both groups achieved spontaneous pregnancy; 45% in the U1a group and 39% in the U2 group delivered at term.

**Conclusions:**

Hysteroscopic metroplasty offers promising reproductive outcomes in the presence of U1a and U2 uterine anomalies for those with unexplained infertility and repeated IVF/ICSI failures. In addition, uterine septum cases should be carefully evaluated intra-operatively to detect and repair concurrent lateral uterine wall anomalies.

## Introduction

The overall prevalence of uterine malformations is 4.7% and may be as high as 25% in patients with a history of miscarriage and infertility (Poncelet and Aissaoui, 2007). Since successful implantation and further development require an appropriate intrauterine environment, uterine malformations are more frequently detected in the context of infertility or repeated miscarriages. The new classification system of uterine anomalies developed by the European Society of Human Reproduction and Embryology (ESHRE) and the European Society for Gynecological Endoscopy (ESGE) recently focussed on this issue (Grimbizis et al., 2013). In this new system, Class U2-septate uterus is the most frequent congenital uterine anomaly. Despite a lack of robust data indicating uterine septum is associated with infertility, evidence suggests that it increases the risk of adverse pregnancy outcomes (Practice Committee of the American Society of Reproductive Medicine - ASRM, 2016). Class U1 incorporates uteri with normal outlines but with an abnormal shape of the uterine cavity excluding septate uteri (T-shaped uteri and tubular-shaped/ infantilis uteri). Although this is a relatively rare condition compared to septate uterus, available data has shown reduced fertility and an increased risk of miscarriage and premature delivery (Chan et al., 2011).

Diagnosis of such uterine anomalies is generally made by hysterosalpingography, three-dimensional (3D) ultrasonography or hysteroscopy. Following the diagnosis, a metroplasty to correct uterine cavity abnormality is the surgical method to improve reproductive and obstetrical outcomes (Ducellier- Azzola et al., 2018).

The principal objective of the current study was to assess the impact of performing metroplasty for T-shaped and septate uteri in women with unexplained infertility or repeated (IVF/ICSI) failures.

## Materials and methods

### 

This was a prospective observational study in a tertiary referral centre between 2015-2018. Patients either with a T-shaped (ESHRE/ESGE U1a) or septate uterus (ESHRE/ESGE U2), and with a history of unexplained infertility or repeated IVF/ ICSI failure (>2 unsuccessful IVF attempts despite the transfer of good quality embryos) were included. Exclusion criteria were body mass index (BMI) >30 kg/m2, secondary infertility, severe male factor infertility, diminished ovarian reserve, a history or diagnosis of endometriosis and/or endometrioma and bilateral tubal obstruction.

### Diagnosis

The diagnosis of a septate or T-shaped uterus was made using a combination of hysterosalpingography, 2D transvaginal ultrasound examination and hysteroscopy. Pelvic magnetic resonance imaging (MRI) was performed in selected cases to confirm the diagnosis.

### Metroplasty Technique

The procedures were scheduled in the early follicular phase of the menstrual cycle under general anaesthesia. Operative hysteroscopy was carried out for all cases by the same surgeon (RP). A bipolar resectoscope and saline distension using a hysteroscopic pump (Endomat; Karl Storz, Tuttlingen, Germany) were used for the procedures. For T-shape uteri, incisions on the lateral walls were made using electroknife (40W) until visualization of both tubal ostia when the hysteroscope was at the internal cervical ostium. For septate uteri, incision of the septum was performed to the level of tubal ostia, additional lateral wall incisions were made if the tubal ostia were not visible with the hysteroscope located at the isthmic level. No additional treatments such as anti-adhesion agents or estrogens were given. All patients were discharged on the day of the procedure. Informed consent was obtained from all patients before the surgical procedure. Institutional Review Board approval was not sought as this was considered as an evaluation of an established service.

### Follow-up

All patients were advised to try to conceive naturally for a period of 12 months. Assisted reproductive technology (ART) was offered to those who had not conceived during this period. Fertility outcomes of all cases were prospectively evaluated. The main outcome parameters were pregnancy and live birth results either achieved spontaneously or with IVF/ ICSI. Clinical pregnancy was defined as a viable fetus with cardiac activity. Miscarriage was defined as the spontaneous loss of a pregnancy prior to the 20th gestational week. Preterm delivery was defined as birth between 20 and 37 weeks of gestation. Term delivery was defined as birth beyond 37 weeks of gestation.

### Statistical analysis

Data analysis was performed using SPSS for Windows, version 22.0 (SPSS Inc., Chicago, IL, United States). Continuous data were described as mean ± SD (standard deviation) for normal distributions, and median (minimum and maximum value) for skewed distributions. Categorical data were described as the number of cases (%). Mean differences between groups were compared by Student’s t test whereas Mann Whitney U test was applied for comparison of median values. Nominal data was analysed by Pearson’s chi-square or Fisher’s exact test, where applicable. A p value less than 0.05 was considered statistically significant.

## Results

A total of 111 cases were included in the analysis. Of these, 48 patients were diagnosed with T-shaped uterus and bilateral longitudinal lateral wall incision was carried out (U1a group). A total of 63 patients were diagnosed with septate or sub-septate uteri (U2). Intra-operatively, uterine septum was confirmed and incised in all cases. In 60 out of 63 septum cases, longitudinal lateral wall incisions were also carried out.

The mean age, BMI and anti-mullerian hormone (AMH) values were 31.7±4, 22±1 and 2.7±1 respectively in the U1a group and 32.2±4, 22.6±1.9 and 2.4±0.9 in the U2 group. The patient characteristics are shown in Table I. Spontaneous pregnancy outcomes following hysteroscopy are shown in Table II. Almost half of patients in each group achieved spontaneous pregnancy (50% and 46% respectively in U1a and in U2 groups). ART outcomes are shown in Table III. There were no cases of cervical incompetence or uterine rupture reported after surgery.

**Table I:** — Patient characteristics of the groups.

	U1a (N=48)	U2 (N=63)	P value
Age (years)	31.7±4.9	32.2±4.8	0.4
BMI (kg/m^2^)	22±1.9	22.6±1.9	0.1
AMH (ng/ml)	2.7±1.5	2.4±0.9	0.5
Additional risk factor	-	-	

NS: not significant; BMI: body mass index; AMH: Anti Mullerian Hormone

**Table II:** — Spontaneous pregnancy outcomes of the groups after metroplasty.

	U1a (N=48)	U2 (N=63)	P value
Spontaneous pregnancy	24 (50% )	29 (46%)	0.6
Mean time to pregnancy (months)	9.2±1.2	8.7±1.6	0.05
First trimester miscarriage	2	1	0.4
Mid-trimester miscarriage	-	1	0.5

NS: not significant

**Table III:** — Assisted reproductive technology outcomes after metroplasty.

	U1a (N=48)	U2 (N=63)	P value
IVF-ICSI	18	17	0.2
Fresh ET	10	11	0.3
Frozen ET	8	6	0.3
Clinical pregnancy/started cycle	10 (55%)	9 (52.9%)	0.8
First trim. miscarriage	2	1	0.5
Mid-trimester miscarriage	1	-	0.2
Delivery between 34-37 weeks	-	-	-

NS: not significant

## Discussion

Our observational study revealed a significant improvement in reproductive outcomes of nulliparous patients either with U1a or U2 uterine anomaly following hysteroscopy. Both spontaneous and IVF/ICSI rates were found to be improved. Interestingly, we noticed that the majority of uterine septum cases revealed lateral wall thickness as well.

Dysmorphic uterus, formerly described as DES related anomaly was recently re-evaluated by a group of ESHRE/ESGE and re-classified as class U1a anomaly (Grimbizis et al., 2013). Moreover, this pathology has been further divided into 3 sub-classes as T shaped, Y shaped and I shaped (tubular) formations (Alonso Pacheco et al., 2019). Despite the controversies on the exact mechanism of dysmorphic uterus and accurate diagnostic methods, current practice favors correcting the uterine morphology to enhance reproductive outcomes (Haydardedeoglu et al., 2018, Di Spiezio Sardo et al., 2019). Improved term delivery rates and decreased miscarriages have been reported by many authors either in small (Garbin et al., 1998) or large cohorts (Fernandez et al., 2011, Ducellier- Azzola et al., 2018, Di Spiezio Sardo et al., 2019). Di Spiezio Sardo et al. (2015) conducted a prospective observational study with 30 infertile women and reported a term delivery rate of 65%. The benefits of metroplasty in patients with dysmorphic uteri have recently been documented in a group of patients with primary infertility, repeated implantation failure and recurrent pregnancy loss (Boza et al., 2019). Recent retrospective data from a multicentre study showed that hysteroscopic correction of dysmorphic uterus may result in high live birth rates in women suffering from unexplained infertility or repeated miscarriages (Di Spiezio Sardo et al., 2019). Similarly, in our cohort, more than half of patients with class U1a delivered at term following metroplasty. It is obvious that, different dysmorphic uterus sub-types require different approaches for surgical correction through hysteroscopic metroplasty and more studies are needed to comment on whether those sub-types may have different reproductive outcomes. However, enlarging lateral walls and correcting the uterine texture in all types seems to increase the endometrial volume and enhance the implantation potential of an embryo.

Septate uterus has been associated with reduced fertility even though the exact mechanism has not been clearly established. Most studies suggest that septum treatment leads to improved spontaneous pregnancy rates in infertile women (Saygılı-Yılmaz et al., 2003, Pabuccu et al., 2004). Moreover, starting an IVF/ICSI cycle immediately after the hysteroscopic procedure does not seem to impair the implantation rate or pregnancy rates compared to those who started later (Berkkanoglu et al., 2008). In our cohort, more than 60% of uterine septum cases conceived either spontaneously or with IVF/ ICSI following metroplasty. Time to pregnancy was shorter than a year and the procedure did not delay time to IVF/ICSI. Moreover, not a single case delivered before 34 week gestation including those with early pre-term delivery history in our cohort. Therefore, we may conclude that metroplasty for uterine septum improves both fertility and obstetrical outcomes.

In our study, we observed lateral uterine wall protrusion in the majority of septum cases. Intraoperatively, bulging of uterine sidewalls restricting clear visualisation of tubal ostia from the level of internal os was seen in these patients. Longitudinal lateral wall incisions were carried out to enlarge the uterine cavity further in addition to division of septum.

The management of unexplained infertility is still controversial. Natural conception rates mostly depend on female age and other fertility factors. Recent data revealed that estimated natural conception rate leading to ongoing pregnancy to be 24.5% after 12 months in couples with unexplained or mild male subfertility scheduled for fertility treatment (Van Ekelen et al., 2018). In our series, the spontaneous pregnancy rates were twice this rate in a group of women with a mean age of 32 years. Moreover, time to pregnancy was nearly 3 months shorter in our dataset. It seems that careful evaluation of the uterine cavity and hysteroscopy offers promising natural conception rates.

Recent international position statements highlighted other potential causes of female infertility due to intracavitary pathologies, such as myomas (Vitale et al., 2019), or chronic endometritis (Cicinelli et al., 2019). When a patient is diagnosed with unexplained infertility, such pathologies should be excluded by using enhanced imaging modalities and hysteroscopy.

While hysteroscopic procedures are widely used to enhance fertility, iatrogenic complications may occur such as perforation, adhesion formation or fluid imbalance. Available reports have not reported major iatrogenic complications, this might be due to the use of experienced surgeons in published reports. Adequate surgical experience is very important to avoid iatrogenic adhesions. In this context, the learning curve of metroplasty for T-shaped uterus may be improved by the use of a virtual reality simulator (Vitale et al., 2019).

Our study has some limitations. We did not include a control group in which the uterine anomaly was not surgically corrected, and the sample size was relatively small. Furthermore, we did not utilise 3D sonography in our center. However, we were able to detect the margins of excessive myometrial tissue in T-shape anomalies mainly based on the sagittal view of high resolution 2D ultrasonography and we used MRI if there were uncertain margins in 2D sonography. We did not encounter any lateral wall perforations during the procedure. Intraoperative appearance of the uterine cavity was an important part of the diagnostic process; if the tubal ostia were not visualized from the level of internal cervical os during operation, lateral wall incisions were performed to improve the size of the uterine cavity.

## Conclusion

In conclusion, we documented enhanced reproductive outcomes after hysteroscopic metroplasty in nulliparous patients with U1a and U2 uterine anomaly and infertility. Particularly for those diagnosed with long standing unexplained infertility and/or repeated IVF/ICSI failures, correcting the uterine morphology seems to enhance both spontaneous and assisted conception pregnancy rates.

## References

[1] Alonso Pacheco L, Laganà AS, Ghezzi F (2019). Subtypes of T-shaped uterus.. Fertil Steril.

[2] Berkkanoglu M, Isikoglu M, Arici F (2008). Fertil Steril. What is the best time to perform intracytoplasmic sperm injection/embryo transfer cycle after hysteroscopic surgery for an incomplete uterine septum.

[3] Boza A, Akin OD, Oguz SY (2019). Surgical correction of T-shaped uteri in women with reproductive failure: Long term anatomical and reproductive outcomes.. J Gynecol Obstet Hum Reprod.

[4] Chan YY, Jayaprakasan K, Zamora J (2011). The prevalence of congenital uterine anomalies in unselected and high-risk populations: a systematic review.. Hum Reprod Update.

[5] Cicinelli E, Vitagliano A, Kumar A (2019). Unified diagnostic criteria for chronic endometritis at fluid hysteroscopy: proposal and reliability evaluation through an international randomized-controlled observer study. International Working Group for Standardization of Chronic Endometritis Diagnosis. Fertil Steril.

[6] Di Spiezio Sardo A, Florio P, Nazzaro G (2015). Hysteroscopic outpatient metroplasty to expand dysmorphic uteri (HOME-DU technique): a pilot study.. Reprod Biomed Online.

[7] Di Spiezio Sardo A, Campo R, Zizolfi B (2019). Long-Term Reproductive Outcomes after Hysteroscopic Treatment of Dysmorphic Uteri in Women with Reproductive Failure: An European Multicenter Study.. J Minim Invasive Gynecol.

[8] Ducellier-Azzola G, Lecointre L, Hummel M (2018). Hysteroscopic enlargement metroplasty for T-shaped uterus: 24 years’ experience at the Strasbourg Medico-Surgical and Obstetrical Centre (CMCO).. Eur J Obstet Gynecol Reprod Biol.

[9] Fernandez H, Garbin O, Castaigne V (2011). Surgical approach to and reproductive outcome after surgical correction of a T-shaped uterus.. Hum Reprod.

[10] Garbin O, Ohl J, Bettahar-Lebugle K (1998). Hysteroscopic metroplasty in diethylstilboestrol-exposed and hypoplastic uterus: a report on 24 cases.. Hum Reprod Oxf Engl.

[11] Grimbizis GF, Gordts S, Di Spiezio Sardo A (2013). The ESHRE/ESGE consensus on the classification of female genital tract congenital anomalies.. Hum Reprod.

[12] Haydardedeoğlu B, Doğan Durdağ G, Şimşek S (2018). Reproductive outcomes of office hysteroscopic metroplasty in women with unexplained infertility with dysmorphic uterus.. Turk J Obstet Gynecol.

[13] Kaufman RH, Adam E (2002). Findings in female offspring of women exposed in utero to diethylstilbestrol. Obstet Gynecol.

[14] Pabuçcu R, Gomel V (2004). Reproductive outcome after hysteroscopic metroplasty in women with septate uterus and otherwise unexplained infertility.. Fertil Steril.

[15] Poncelet C, Aissaoui F (2007). Uterine malformations and reproduction. Gynécol Obstétr Fertil.

[16] (2016). Uterine septum: a guideline.. Fertil Steril.

[17] Saygili-Yilmaz E, Yildiz S, Erman-Akar M (2003). Reproductive outcome of septate uterus after hysteroscopic metroplasty.. Arch Gynecol Obstet.

[18] van Eekelen R, Tjon-Kon-Fat RI, Bossuyt PMM (2018). Natural conception rates in couples with unexplained or mild male subfertility scheduled for fertility treatment: a secondary analysis of a randomized controlled trial.. Hum Reprod.

[19] Vitale SG, Sapia F, Rapisarda AMC (2017). Hysteroscopic morcellation of submucous myomas: a systematic review.. Biomed Res Int.

[20] Vitale SG, Caruso S, Vitagliano A (2019). The value of virtual reality simulators in hysteroscopy and training capacity: a systematic review.. Minim Invasive Ther Allied Technol.

